# Newly discovered populations of the Ethiopian endemic and endangered *Afrixalus clarkei* Largen, implications for conservation

**DOI:** 10.3897/zookeys.565.7114

**Published:** 2016-02-17

**Authors:** Jan Mertens, Merlijn Jocqué, Lore Geeraert, Matthias De Beenhouwer

**Affiliations:** 1 BINCO vzw. Biodiversity Inventory for Nature Conservation, 3380 Glabbeek, Belgium; 2 Plant Conservation and Population Biology, Biology Department, KU Leuven, Kasteelpark, Arenberg 31, 3000 Heverlee, Belgium; 3 Aquatic and Terrestrial Ecology (ATECO), Royal Belgian Institute of Natural Sciences, Vautierstraat 29, 1000 Brussels, Belgium

**Keywords:** Amphibians, Distribution, Forest, IUCN, Survey, Southwest Ethiopia

## Abstract

Knowledge of the Ethiopian amphibian fauna is limited and Southwest Ethiopia remains understudied. This part of Ethiopia, where most of the country’s remaining natural forest is situated, is known to harbour the only populations of *Afrixalus clarkei* (Largen), an endemic banana frog, worldwide. This species is under great threat of extinction and is therefore classified as endangered on the IUCN red list. We surveyed different potential habitats for this species outside its known range and found several new populations extending its known habitat preference, and the geographical and altitudinal range of the species. We here show that *Afrixalus clarkei* is more common than previously thought.

## Introduction

The highlands of Ethiopia are characterized by a high endemism of fauna and flora (Evangelista et al. 2008; Freilich et al. 2014), and acknowledged as one of the prime biodiversity hotspots globally (Mittermeier et al. 2011). Despite a recent surge in amphibian studies (Weinsheimer et al. 2010; Mengistu 2012), including several expeditions to the undulating highlands (Gower et al. 2012; Freilich et al. 2014), substantial knowledge gaps on the Ethiopian amphibian fauna remain. Based on Amphibiaweb (2015), 66 species of amphibians are currently recorded from Ethiopia (see also Largen 2001), of which 41% are endemic (Evangelista et al. 2008; AmphibiaWeb 2015) and 38 species are known to occur in Southwest Ethiopia (Largen and Spawls 2010). Still, the Southwest of Ethiopia remains poorly documented with data from only two herpetological expeditions (Largen 1974; Gower et al. 2013) together with some sporadic observations (summarized in Largen 2001). Although the Southwest of Ethiopia is known to harbour the last large tracts of natural forest, forest cover has declined dramatically to less than 3% nationwide (Dessie and Christiansson 2008). Therefore, accurate information on species conservation and distribution is an essential first step to facilitate the delivery of conservation updates, recognize biodiversity hotspots and encourage habitat protection and restoration (De Beenhouwer et al. 2015a; Rovero et al. 2014).

## Materials and methods

The authors conducted fieldwork in the Jimma zone, Oromia region, in Southwest Ethiopia. Within the Jimma zone, the Belete-Gera forest is an evergreen montane forest that ranges up to 3000 m a.s.l. and has considerable cover of moist evergreen montane forest. In August 2014, the middle of the rainy season, we completed an assessment of the amphibian fauna in one of the largest remaining natural forest tracts in the area around Afalo (7°38.02'N; 36°13.17'E) between 1600 and 2200 m a.s.l. (De Beenhouwer et al. 2015b). We used both visual encounter survey methods and pitfall trapping to assess the amphibian diversity in the forest (Rödel and Ernst 2004). Identification was based on morphology (Largen 2001, amongst others).

## Results and discussion

Amphibians were searched for by the team members on ten evenings in August 2014, resulting in 111 search hours across seven different locations. In total, 13 amphibian taxa were identified from our surveys (Table 1). The most common species were *Hyperolius viridiflavus* (Dumeril & Bibron, 1841) and *Phrynobatrachus minutus* (Boulenger, 1895), accounting for approximately 48% of the species surveyed. Thirty-eight percent of the identified species were endemic to Ethiopia (Table 1). *Hyperolius kivuensis* (Ahl, 1931) was observed in two locations around Afalo on the 21^st^ and 22^nd^ of August. This species, listed as ‘Least Concern’ (IUCN 2015.2), is shown here to extend its range with approximately 150 km to the East of the country (IUCN 2013b). All species identified are listed as ‘Least Concern’ on the IUCN red list, except for *Afrixalus clarkei* (Largen, 1974), which is considered ‘Endangered’ (B1 ab(iii); IUCN 2012.2).

**Table 1.** List of amphibian species found in the Belete-Gera forest during the August 2014 survey. The asterisk (*) indicates the species that are new for the area, Ethiopian endemic speies are followed by (E). #ind. = minimum number of individuals encountered. IUCN-status EN = Endangered, LC = Least concern.

Species	#ind.	IUCN-status (2014)
Afrixalus clarkei* (E)	100	EN
*Amietophrynus asmarae* / *regularis*	20	LC
*Conraua beccarii* Boulenger	20	LC
*Hemisus microscaphus* Laurent (E)	20	LC
*Hoplobatrachus occipitalis* Günther	5	LC
Hyperolius kivuensis*	10	LC
*Hyperolius viridiflavus*	100	LC
*Leptopelis vannutellii* Boulenger (E)	50	LC
*Paracassina obscura* Boulenger (E)	100	LC
*Phrynobatrachus minutus* (E)	100	LC
*Phrynobatrachus natalensis* Smith	50	LC
*Ptychadena* spp.	100	
*Xenopus clivii* Peracca	20	LC

**Figure 1.**
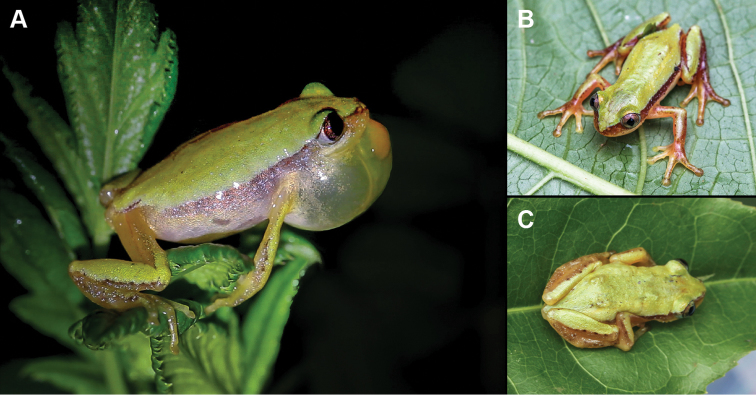
*Afrixalus clarkei*; calling male (**A**), frontal view of male (**B**), dorsal view of female (**C**). Pictures: J. Mertens.

*Afrixalus clarkei*, an Ethiopian endemic frog (Fig. 1), was recorded from the banks of the Kito river South of Jimma (10 August 2014, 7°40.08'N; 36°49.12'E, 1722 m a.s.l.), in a swamp in the floodplain of a river South of Chira (14 August 2014, 7°40.08'N; 36°14.56'E, 2030 m a.s.l.), and in the moist montane evergreen forest around Afalo (16 August 2014, 7°38.01'N; 36°13.16'E, 1829 m a.s.l. and 20 August 2014, 7°37.09'N; 36°13.48'E, 1784 m a.s.l.). Most specimen had a plain green dorsum and brown dorsolateral lines fading towards the back (Fig. 1; Largen and Spawls 2010), one male in Afalo had an overall turquoise dorsum. Adult males in our sampling reached a maximum snout vent length (SVL) of 23 mm (avg. length 20.3 mm, avg. weight 0.52 g), the largest female reached 24.3 mm SVL (avg. length 23.2 mm, avg. weight 0.71 g).

**Figure 2.**
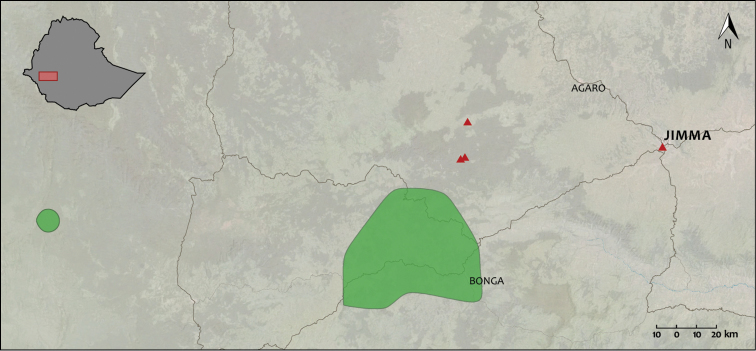
Distribution map of *Afrixalus clarkei*. Green polygons represent previously known distribution. Red triangles represent new records.

*Afrixalus clarkei* was previously only known from two populations in Southwest Ethiopia between 820 and 1800 m a.s.l. in moist tropical forest near Bonga (Largen and Spawls 2010; Gower et al. 2012). Our findings extend the distribution of *Afrixalus clarkei* by roughly 40 km towards the North (Chira) and 70 km to the East (Jimma) (Fig. 2; IUCN 2013a). It also extends the altitudinal range to a maximum of 2030 m a.s.l. (Chira). Our observations of *Afrixalus clarkei* outside forest habitats, in marshes and riverine floodplains in open disturbed landscapes, suggest a higher degree of tolerance against forest degradation than previously expected. The populations found in floodplains along the Kito River in Jimma and the Naso River in Chira suggest that the species still has a larger distribution than currently documented. These observations illustrate the limited knowledge on amphibian distribution and conservation in this part of Ethiopia.
